# Protective effect of resveratrol on TNF-α-treated vascular endothelial cells

**DOI:** 10.1186/1479-5876-10-S2-A59

**Published:** 2012-10-17

**Authors:** Jingyu Xu, Yuan Lin, Ping Fu, Li Wang, Hua Li

**Affiliations:** 1 Department of Pharmacology, College of Pharmacy, Dalian Medical University, Dalian City, Liaoning Province, 116044, China

## Objectives

Thrombomodulin (TM) is an important index of vascular endothelial injury. The present study was designed to investigate the effects and mechanims of resveratrol (RES) on TM in TNF-α-injured vascular endothelial cell (VEC), and thus to provide a reliable experimental basis of RES for the endothelial function improvement and cardiovascular disease prevention.

## Methods

1. VEC was treated with different concentration of TNF-α (0, 1.25, 2.5, 5, 10 and 20ng/ml) for 4 hours. Then the content of NO and the activities of iNOS, and eNOS in the cell medium and lysates of VEC were determined, and the expression of TM was analyzed by using Western blot.

2. VEC was incubated with various concentrations of RES for 2 hours before adding 10ng/ml TNF-α. Then the content of NO, the activities of iNOS and eNOS were measured. The expression of TM was analyzed, and the activity of TM was determined by using thrombin time (TT) detection method.

3. VEC was incubated with various concentrations of SIRT1 inhibitor nicotinamide for 30min before adding RES and TNF-α.

## Results

1. TNF-α increased the production and release of NO, the activities of iNOS, and TNF-α decreased the activity of eNOS significantly. It also reduced the expression of TM. Moreover, the optimal concentration of TNF-α for the injury of VEC was 10ng/ml.

2. Compared with TNF-α-injured group, all RES treatments decreased significantly the content of NO,the activity of iNOS, increased the activity of eNOS, and improved the expression and the activity of TM significantly.

3. Nicotinamide showed significantly inhibitory effects on the protective effects of RES.

## Conclusion

1. VEC injury model was established successfully with TNF-α treatment.

2. RES showed significant protective effects on TNF-α-injured VEC, including the recovery of NO system, the increase of the expression and the activity of TM.

3. Nicotinamide inhibited the protective effects of RES, suggesting that this protective effect of RES be related to SIRT1 pathway.

